# Violent assault geographies in northeastern Australia

**DOI:** 10.1371/journal.pone.0282522

**Published:** 2023-03-02

**Authors:** Tarah Hodgkinson, Jonathan Corcoran, Martin A. Andresen

**Affiliations:** 1 Department of Criminology, Wilfrid Laurier University, Brantford, Ontario, Canada; 2 School of Earth and Environmental Sciences, University of Queensland, Brisbane, Queensland, Australia; 3 School of Criminology, Simon Fraser University, Burnaby, British Columbia, Canada; Chinese Academy of Sciences, CHINA

## Abstract

As climate change produces more extreme weather, it is increasingly important to understand the impacts of these changes on social behaviour. The relationship between weather and crime has been studied across numerous contexts. However, few studies examine the correlation between weather and violence in southern, non-temperate climates. In addition, the literature lacks longitudinal research that controls for international changes in crime trends. In this study, we examine over 12 years of assault-related incidents in the state of Queensland, Australia. Controlling for deviations in trend for temperature and rainfall, we explore the relationship between violent crime and weather across Köppen climate classifications. Findings provide important insight into the impact of weather on violence across temperate, tropical, and arid climate regions.

## Introduction

Climate change is creating weather extremes around the world [1]. These weather extremes not only impact the health and well-being of people, but also their social behaviour. The literature on the connection between weather and crime is extensive [2–8]. However, much of this research has been conducted in the global north and examines climate zones that are generally warm/hot in the summer and cool/cold in the winter. Apart from a few studies conducted in subtropical and tropical climates [9–11], little is known about the influence of other climate regions on social behaviour, that impact on crime over time, and across diverse climate regions.

The impact of climate change on social behaviour, particularly crime, needs to be better understood. As weather intensifies the potential impact on community safety could be tremendous. The literature offers three main explanations for the impact of weather on crime: temperature-aggression, heat reclusion, and routine activities. Temperature-aggression theory suggests that as heat increases, so too do aggressive actions [12–14]. Heat reclusion theory also finds an initial linear relationship between heat and crime, but this relationship inverts when temperatures exceed a certain point and people seek shelter from the weather/heat [13]. Finally, proponents of the routine activities perspective would argue that changes in weather create different opportunities for crime, as suitable targets and motivated offenders are more or less likely to come together in time and space [15–19]. Of course, temperature is not the only weather type. Some studies have also linked precipitation levels to crime [20, 21]. Few studies look at the impact of both of these explanatory mechanisms in nontemperate zones—there are some notable exceptions [9, 16, 17, 22].

This study fills a gap in the literature by examining the impact of different weather extremes—defined here as significant deviations from temperate and rainfall averages—on violence across the State of Queensland located in Northeastern Australia. Queensland covers seven Köppen climate zones: *Arid steppe hot*; *Temperate*, dry winter, hot summer; *Temperate*, no dry season, hot summer; *Tropical monsoon*; *Tropical rainforest*; *Tropical savanna*, dry winter; and *Temperate*, no dry season, warm summer. However, because of the small physical area (less than 0.2 percent of the study area), and low counts of violent crime, covered by *Temperate*, no dry season, warm summer and *Tropical rainforest*, these Köppen classifications were recoded to *Temperate*, dry winter, hot summer and *Tropical monsoon*, respectively: *Temperate*, no dry season, warm summer is slightly inland and surrounded by *Temperate*, dry winter, hot summer; *Tropical rainforest* is immediately north of *Tropical Monsoon* and both are coastal regions. Despite the need to perform these aggregations, this allows for a wide range of meaningful comparative analysis of the weather-crime relationship. We also examine these relationships, and deviations from trend, across 12 years of data at the daily level. This longitudinal analysis allows us to account for any deviations from normal or expected crime levels that can be explained through unusual or exceptional weather events as well as any short-term changes due to policy implementation. Examining the role of weather extremes on social behaviour in a non-temperate climate not only provides an opportunity to test these theories of weather and crime in non-temperate climates, but also provides guidance on how to adapt crime prevention policy and practice in preparation for more extreme weather events in the future.

## Related literature

The relationship between weather and social behaviour is a longstanding area of research [6, 23, 24]. Beginning in the 1800s, Adolphe Quetelet [25] explored the link between seasons and crime, finding that violence increased in the summer months and property crime in the winter. Since then, researchers have continued to find connections between ‘good’ weather and increases in crime [26, 27]. However, much of this research has explored temperate climates that experience four distinct seasons. This leaves a theoretical and empirical gap in creating policy and prevention efforts for non-temperate climate zones as less is known about how deviations in weather in these zones, or “weather shocks” [28] affect daily crime patterns.

### Theoretical relationships between weather, climate, and crime

As outlined briefly above, there are three main theoretical explanations related to weather, climate, and violence. These theories focus on the impact of temperature and offer a linear, u-shaped, or opportunity dependent explanation of aggression. *Temperature aggression theory* suggests a linear relationship between temperature and crime. As temperature increases, so do aggressive actions because increases in heat lead to increases in aggression and hostility [13, 29–32]. Temperature aggression theory has been critiqued, arguing that the relationship is only linear until the point of discomfort. *Heat reclusion theory*, also known as the *negative affect escape* model, suggests a u-shaped, or curvilinear, pattern between heat and aggression. Proponents of this theory argue that the relationship between heat and aggression is linear to a point. However as increases in heat cause discomfort, violence or crime reduces, because people will retreat and seek relief from discomfort [23, 33]. This theory has also received some criticism, as it is difficult to test non-linearity in areas with relatively consistent weather patterns or poorer areas without access to sources of relief like air conditioning [10].

The *routine activities* perspective argues that behavioural patterns change in response to changes in temperature, not because of the temperature itself, but because of the opportunities those temperatures create. For example, in the summer, when heat increases, individuals are more likely to spend time outside and engaging with others (particularly in in temperate climates). As the opportunities for motivated offenders and suitable targets to converge in time and space increases, so too do opportunities for crime [34–36]. Previously, empirical studies that have also controlled for day of the week, or public holidays, have found that rates of violence and homicide were higher on weekends and rates of interpersonal violence were higher on public holidays [10, 17, 37]. They argue that as individuals have more free time, this creates opportunities for violence, suggesting that heat acts as a predictor of opportunity that then predicts violence or aggression. Furthermore, the routine activity perspective often provides a better explanation for deviations in weather across place and time as these patterns change depending on location and day of the week or time of day [20, 38]. Considering that the routine activities perspective explains both predicted patterns in temperature aggression theory and heat reclusion theory, while also accounting for the impact of other weather types, we adopt this framework to interpret the findings in the current study.

### Heat, precipitation, and crime

The evidence for the connection between how weather impacts routine activities, or opportunities, and crime is strong. This is true for both heat as well as precipitation. As discussed, researchers have long found that violence increases as temperature increases and people spend more time outside, away from the home and in the presence of others [39–41]. For example, in Philadelphia, violence and social disorder were at their highest on days with pleasant weather (between 22 and 28 degrees Celsius) [42]. Furthermore, daily ambient temperature impacted assault (both simple and aggravated), and that these impacts were stronger on weekends [43]. According to the routine activities perspective, this is unsurprising because people generally have more unstructured time on weekends, when they are not working or in school [44, 45].

The research on the impact of precipitation (or rainfall), on crime is less extensive than that on temperature. Furthermore, while the routine activities perspective would suggest that precipitation is more likely to push people inside and away from potential crime opportunities [46], much of this research provides mixed results depending on crime type. The impact of precipitation on assault and violence appears to be consistently negative. For example, in the United States, increases in weekly precipitation produced overall reductions in violence [28]. In New Zealand, the relationship between precipitation and violence is negative, but there is a positive relationship between precipitation and property crime [7].

When examining the impact of precipitation on property crime, studies have found contrasting results. For example, a negative relationship was found between rainfall and theft in Singapore [47]. However, in 19^th^ Century Germany, the impact of drought on food prices led to more property crime and less violent crime [48]. More recently, in a study that explored the impact of precipitation on crime over 30 years using monthly data in the United States found that precipitation led to increases in burglary and vehicle thefts [8]. Proponents of the routine activities perspective would suggest this difference in pattern for violent as compared to property crime, is likely a result of the added coverage and reduced visibility and surveillance provided by precipitation [49, 50].

Interestingly, some studies find no connection between precipitation and crime at all [21, 51]. For example, no association between several crime types and precipitation levels was found in England and Wales [52], and in Los Angeles no link between precipitation and violent crime was found [53]. The research on precipitation and crime is quite mixed. It is possible that these mixed results are a product of aggregation. For example, seasonality patterns could actually be affecting the relationship between weekly or monthly precipitation data and crime [54, 55]. This has also been shown recently comparing daily and hourly crime counts [56]. This emphasizes the importance of being able to compare not only across different measures of time, but also across regions that experience different climate regimes.

### Non-temperate climate regions and the impact of weather on crime

The impact of weather, specifically precipitation, differs across climates regions. Non-temperate climate regions are characterised by narrower temperature ranges and generally experience more variable levels of precipitation. This climate dynamic has the potential to create an issue in deriving a clear relationship between weather and violence. Also, certain climate regions create weather norms that are subjectively experienced by those living in such locales. For example, Canadians living in the north of the country have adapted their lifestyles to exists in conditions that have temperatures that are consistently well below freezing, while those living closer to the equator have likely adapted and prepared for higher temperatures or storm seasons [46, 57]. Of course, this will also depend on what resources are available to residents of these different climate regions, including infrastructure such as air conditioning and building quality, to remain cool and dry throughout annual climate cycles. As such, it is important to compare these findings across several regions in the similarly resourced areas, like a State or country, to allow for more robust comparisons to be made across different situational contexts.

In Baranquilla, Colombia, an increase in precipitation of 10mm was associated with a 2% decrease in interpersonal violence [10]. They also found a weak linear relationship between temperature and interpersonal violence. However, they were unable to identify a statistically significant association between weather and homicide. In an examination of deviations from historical weather in non-temperate India, deviations (or weather shocks) in precipitation led to increases in both property and surprisingly violent crime [58]. In the tropical region of Ibadan, Nigeria, research examining assault, burglary, and robbery found that the rainy season to an increase in robberies, while assault and burglary were more common in the dry season [59]. However, all of these studies explore poorer regions. It is important to examine these patterns across areas with greater access to resources as well.

In Taiwan, examining extreme precipitation, specifically typhoons that produce lower temperatures and heavy rain, research has found that typhoons produce an initial decrease in crimes such as violence and vehicle theft, followed by an increase (or lagged effect) in crime [11]. Typhoons that last longer than usual increase rates of violence, theft of vehicle, and robbery, but reduce rates of burglary. The authors suggest these findings are likely a result of poor visibility produced by medium-intensity typhoons that impacts the number of capable guardians but creates more guardianship over people’s homes as they shelter in place. However, the authors do not appear to control for seasonality, which may produce counter findings.

In the context of subtropical Brisbane, Australia, research has explored the impact of weather on assault. In this case, the authors control for seasonality, as well as a range of demographic factors, and found that assaults occurred more often in the spring than the summer in this climate region [60]. They also examined daily trends and found that assaults increased on weekends in comparison to weekdays. These findings are consistent with the daily fluctuations of crime, but indicates that in a subtropical climate, temperature may have slightly different affects on crimes like assault, despite the criminal opportunities provided by unstructured summer activities.

## Current study

As discussed above, much of the work on weather and crime has examined temperate climates. Temperate climates such as Canada, the United States, and England and Wales, experience significant changes in temperature across the four seasons. However, non-temperate climates, typically located closer to the equator, do not experience as much diversity in weather across the annual cycle. It stands to reason that individuals living in these areas are less likely to be impacted by the offending opportunities presented by pleasant weather, if the weather is consistently warm [60]. This has important implications for theory. It calls into question at which level do weather changes in non-temperate zones create opportunities for violence or aggression? Is it significant deviations from the typical weather pattern that create new opportunities? Or, are non-temperate climates more impacted by non-weather related opportunities? For example, is it simply day of the week, or holidays, that create opportunities? Or is it the first sign of change in weather, like that of spring [60]?

As noted, some initial research in non-temperature climates provides initial insight into these questions. In the subtropical climate of Taiwan, research has found that climate-change related increases in temperature led to increases in all violent crime crimes [11]. In the subtropical climate of Sao Paulo, Brazil, homicide was impacted by weather variables, but variables representing changes in routine activities are stronger predictors [17]. In the torrid climate zone of Barranquilla, Colombia, while weather did not impact certain violent crimes like homicide, it did predict interpersonal violence [10]. These findings suggest that as weather becomes more extreme in a non-temperate climate, opportunities for violence may increase. However, this research largely focuses on poorer nations and is often confined to the city level and one climate zone.

In our study, we examine the impact of weather deviations on violent crime across several climate zones and over 12 years in Queensland, Australia. As noted above, the state of Queensland has seven Köppen climate zones that are aggregated into five climate zones. This allows us to explore more than just the dichotomy between temperate and non-temperate, but also across other climate types. There are also some opportunities for comparison with previous research here. Like Baranquilla, Colombia [10], Queensland is generally warm and humid. However, unlike Baranquilla, Queensland is located within the country of Australia, a wealthy nation with relatively low rates of crime and violence (https://www.numbeo.com/crime/rankings_by_country.jsp). Thus, findings may be more likely to mimic those of found in Taiwan [11]. However, this may only be the case for certain climate zones.

The current study makes three key contributions. First, we provide a longitudinal analysis of weather and violent crime that controls for the international crime drop. This is important considering that much of this work has not accounted for this large-scale phenomenon [61–65]. We also control for seasonality [41] and days of the week [60] as these impact opportunities for crime are not always included in similar studies. Second, we use several Köppen climate regions to guide our climate analysis. Most studies only examine one climate region (specifically one city within one climate region), making the results difficult to generalize to other areas. In this study, we analyze five climate regions, allowing us to test current theoretical explanations both within, and across, Queensland, Australia. Third, we use a measure of daily deviations from the trend in temperature and precipitation. These daily deviations are more exact and better reflect the changes in behaviour according to changes in temperature and precipitation. We hypothesize that routine activities will be impacted by significant deviations in weather norms, and that extreme heat or rainfall will reduce opportunities for interaction between offenders and victims, thus reducing rates of violent crime.

## Data and methods

### Study area

**Fig 1:**
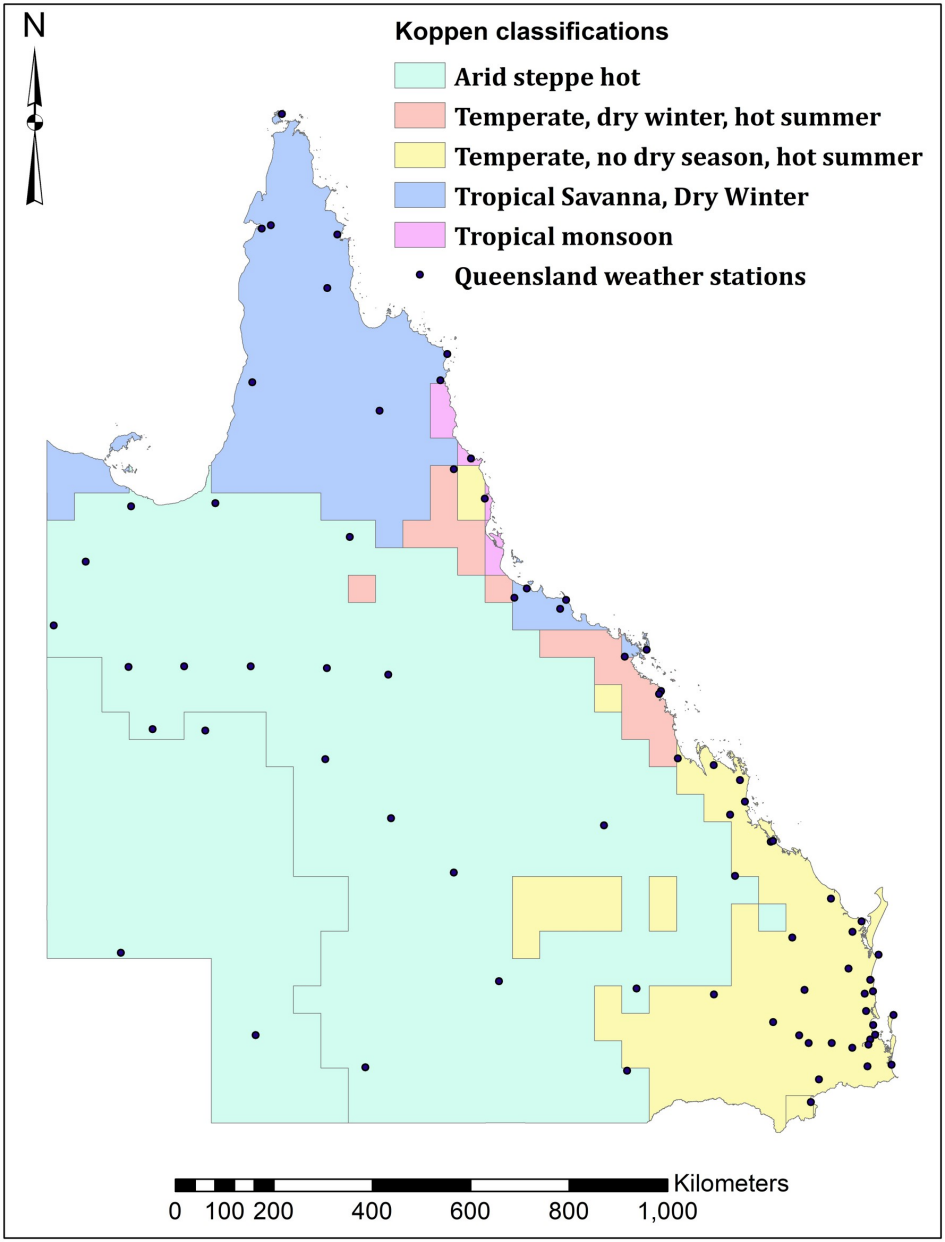
Köppen classifications, Queensland, Australia.

We analyse violent crimes across the entire state of Queensland, Australia. Queensland is approximately 1.85 million square kilometers (about 14 times larger than England) with a population of approximately 5 million people (less than one tenth the population of England), resulting in a fairly low population density outside of urban areas like Brisbane and the Gold Coast. Queensland is separated into 5 Köppen classifications (regions) of varying sizes, see Fig 1—Köppen classification data are freely available from the World Bank Data Catalog [66], with Queensland weather stations freely available from Australian government’s Bureau of Meteorology [67], and the Queensland coastline and state border freely available from the Queensland Government Open Data Portal [68]. Based on area, the majority of Queensland is classified as Arid steppe hot (between desert and humid); based on population, the majority is Queensland is classified as Temperate, no dry season, hot summer (Brisbane and the Gold Coast) with approximately one-half of Queensland’s population.

### Data

Queensland contains the following Köppen classifications: *Arid steppe hot*; *Temperate*, dry winter, hot summer; *Temperate*, no dry season, hot summer; *Tropical monsoon*; *Tropical rainforest*; *Tropical savanna*, dry winter; and *Temperate*, no dry season, warm summer. However, because of the small geographic area low violent crime counts, for *Temperate*, no dry season, warm summer and *Tropical rainforest*, these Köppen classifications were recoded to *Temperate*, dry winter, hot summer and *Tropical monsoon*, respectively. These two recoded Köppen classifications comprise less than 0.2 percent of the entire land area of Queensland and, as noted above, are geographically contiguous to the regions they are aggregated to: *Temperate*, no dry season, warm summer is slightly inland and surrounded by *Temperate*, dry winter, hot summer; *Tropical rainforest* is immediately north of *Tropical Monsoon* and both are coastal regions. The separation of Queensland into these climate regions, or classifications, allows for us to investigate the impact of weather on crime across different climactic condition geographies across the entire state.

We analyze violent crime and weather data from 01 January 2008 through to 31 December 2019 (n = 4,380). As such, we analyze 12 years of daily count data and do not consider 2020 forward to avoid any complicating aspects of COVID-19 for our research design [69, 70]. All violent crime data are obtained from Queensland Police Service though the Griffith Criminology Institute’s Social Analytics Laboratory at Griffith University. Violent crime types include assault, robbery, sexual assault, and fighting. All climate data are obtained through the Australian government’s Bureau of Meteorology [67]. Rainfall (in millimeters) and temperature (daily maximum) data are calculated from the 76 weather stations operating across Queensland for the entire study period—all weather stations are shown on Fig 1. All Köppen classifications had more than one weather station except for Tropical monsoon, because of its small size. Rainfall and temperature data (trend and deviation from trend, both calculated using the Hodrick-Prescott filter with the trend component incorporating lagged values) used in the analyses represent the average values for all weather stations in each Köppen classification. We also included squared terms for rainfall and temperature to account for nonlinearities, but they we never statistically significant so they were removed from the final results presented below.

The descriptive statistics for the violent crime counts are shown in Table 1. *Temperate*, no dry season, hot summer has the highest average, minimum, and maximum counts. However, this is simply because half the population of Queensland resides in this area (Brisbane and Gold Coast). This Köppen classification accounts for approximately 33 percent of violent crime in Queensland, that does leave two-thirds of the violent crime counts to be present in the other 4 Köppen classifications. As such, we do not have any concerns for lack of variation in violent crime counts for our analyses. There is meaningful variation in the average rainfall levels in each of the Köppen classifications—trend and cycle data are shown for the interested reader but are not discussed here. *Arid steppe hot*, that is the majority of the Queensland outback, is, not surprisingly, the driest region in Queensland. This is followed by: *Temperate*, dry winter, hot summer; *Temperate*, no dry season, hot summer; *Tropical savanna*, dry winter; and *Tropical monsoon*. With regard to maximum rainfall *Tropical monsoon* (Cairns, a popular tourist region) has almost 5 times the maximum rainfall than *Temperate*, no dry season, hot summer (Brisbane and Gold Coast, the most populous region). There is less variation in temperature values across Queensland, but Arid steppe hot and Tropical monsoon are the hottest regions in Queensland.

**Table 1:** Descriptive statistics, by climate region.

Variable	Mean	Std Dev.	Median	Minimum	Maximum
Arid steppe hot (ASH), violence	2.5	2.16	2	0	18
Temperate, dry winter, hot summer (TDWHS), violence	1.27	1.46	1	0	27
Temperate, no dry season, hot summer (TNDSHS), violence	27.62	10.83	26	5	225
Tropical monsoon, (TM), violence	2.73	2.28	2	0	39
Tropical savanna, dry winter (TSDW), violence	4.95	3.58	4	0	88
ASH, rain	1.37	3.3	0.04	0	39.23
ASH, rain trend	1.37	0.85	1.25	0.09	3.71
ASH, rain cycle	0	3.05	-0.65	-3.7	37.22
TDWHS, rain	3.85	11.53	0.07	0	180.3
TDWHS, rain trend	3.85	2.5	3.23	0.65	15.75
TDWHS, rain cycle	0	10.98	-2.06	-15.21	168.97
TNDSHS, rain	3.01	6.69	0.7	0	100.85
TNDSHS, rain trend	3.01	1.26	2.76	0.92	7.23
TNDSHS, rain cycle	0	6.45	-1.72	-6.94	95.27
TM, rain	5.62	20.56	0	0	474
TM, rain trend	5.62	3.41	4.81	-0.17	19.42
TM, rain cycle	0	19.93	-3.29	-19.42	461.19
TSDW, rain	4.06	7.81	0.59	0	90.72
TSDW, rain trend	4.06	2.65	3.61	0.3	17.12
TSDW, rain cycle	0	6.74	-1.56	-13.2	85.04
ASH, temperature	31.91	5.34	32.54	12.51	43.63
ASH, temperature trend	31.91	2.56	31.86	27.47	40.8
ASH, temperature cycle	0	3.43	0.46	-15.67	8.21
TDWHS, temperature	27.88	3.24	28.1	18.3	39.23
TDWHS, temperature trend	27.88	1.54	27.89	24.98	32.78
TDWHS, temperature cycle	0	2.09	0.06	-7.05	9.42
TNDSHS, temperature	26.64	3.89	26.89	14.85	38.56
TNDSHS, temperature trend	26.64	1.83	26.62	23.36	32.6
TNDSHS, temperature cycle	0	2.53	0.04	-9.5	9.15
TM, temperature	29.59	2.51	29.7	20.3	42.6
TM, temperature trend	29.59	1.08	29.6	27.57	32.38
TM, temperature cycle	0	1.79	0.12	-7.59	11.42
TSDW, temperature	30.25	2.22	30.39	21.99	38.58
TSDW, temperature trend	30.25	1.06	30.25	28.21	34.19
TSDW, temperature cycle	0	1.45	0.11	-7.63	6.95

In addition to these climate-based variables, we include a number of control variables in our analyses. These include: a linear trend, month and month-squared, day and day-squared (day of the year, 1 to 365), and a dummy variable for each day of the week with Sunday as the base. The linear trend accounts for the international crime drop [61–65], the month and day variables account for known seasonality in crime data [15, 22, 41, 71–74], and the day of the week dummy variables account for known changes in (violent) crime volumes throughout the week [75–77].

## Methods

Because of the nature of the crime data (violent crime counts), a count data model is appropriate. Due to overdispersion identified in the data, only negative binomial models are estimated. Negative binomial models are used in two common specifications: standard and zero-inflated—truncated/hurdle specifications are also available but less common. Standard negative binomial models estimate counts of events, with lower valued counts often expected to be more frequent. Zero-inflated negative binomial models consider zeros to come in two forms, regular zeros and excess zeros. Regular zeros are a component in a standard negative binomial model, but excess zeros are zeros that are qualitatively different.

In a criminological context, these may occur in areas that never have criminal events (excess zeros) and areas that may have no criminal events. This is most often found when modeling specific crime types that may only occur in particular places. For example, commercial burglary can only occur where there is commercial or mixed land use. These negative binomial models generate output that have two components: one to model the excess zeros and one for the other data. Though there is no reason, a priori, to assume such a situation in our research context, we conducted the Vuong test to identify if a zero-inflated model is a better fit to the data than a standard negative binomial regression model. In all cases, the Vuong test did not identify statistically significant reductions in AIC values. As such, the standard negative binomial model is used for all Köppen classifications here. All statistical analyses are performed with The R Project for Statistical Computing, using the *pscl* library [78] and we use heteroskedastic-autocorrelation consistent standard errors for all inference.

## Results

**Fig 2:**
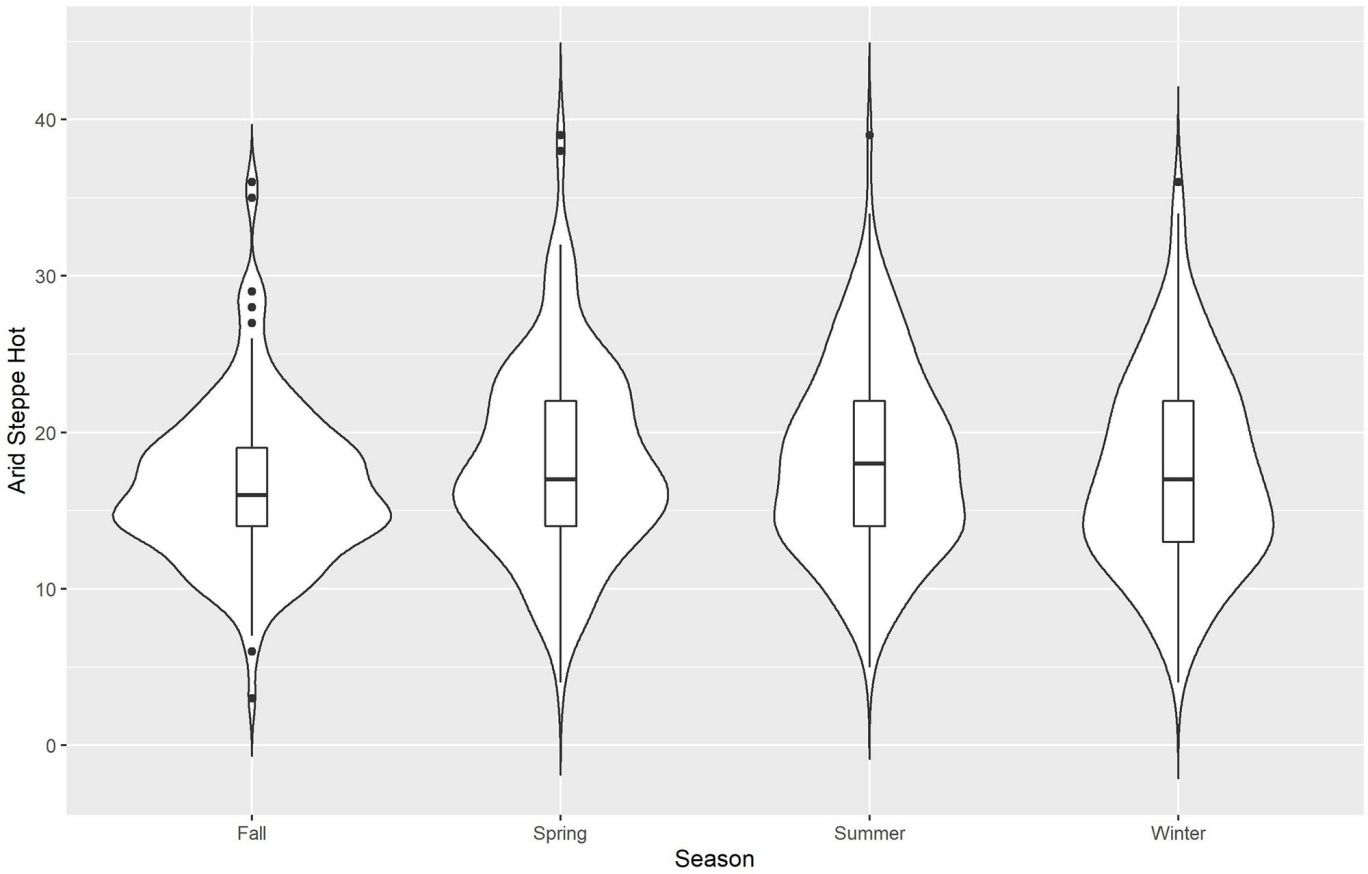
Violin plots for violent crime counts, by season: Arid steppe hot.

**Fig 3:**
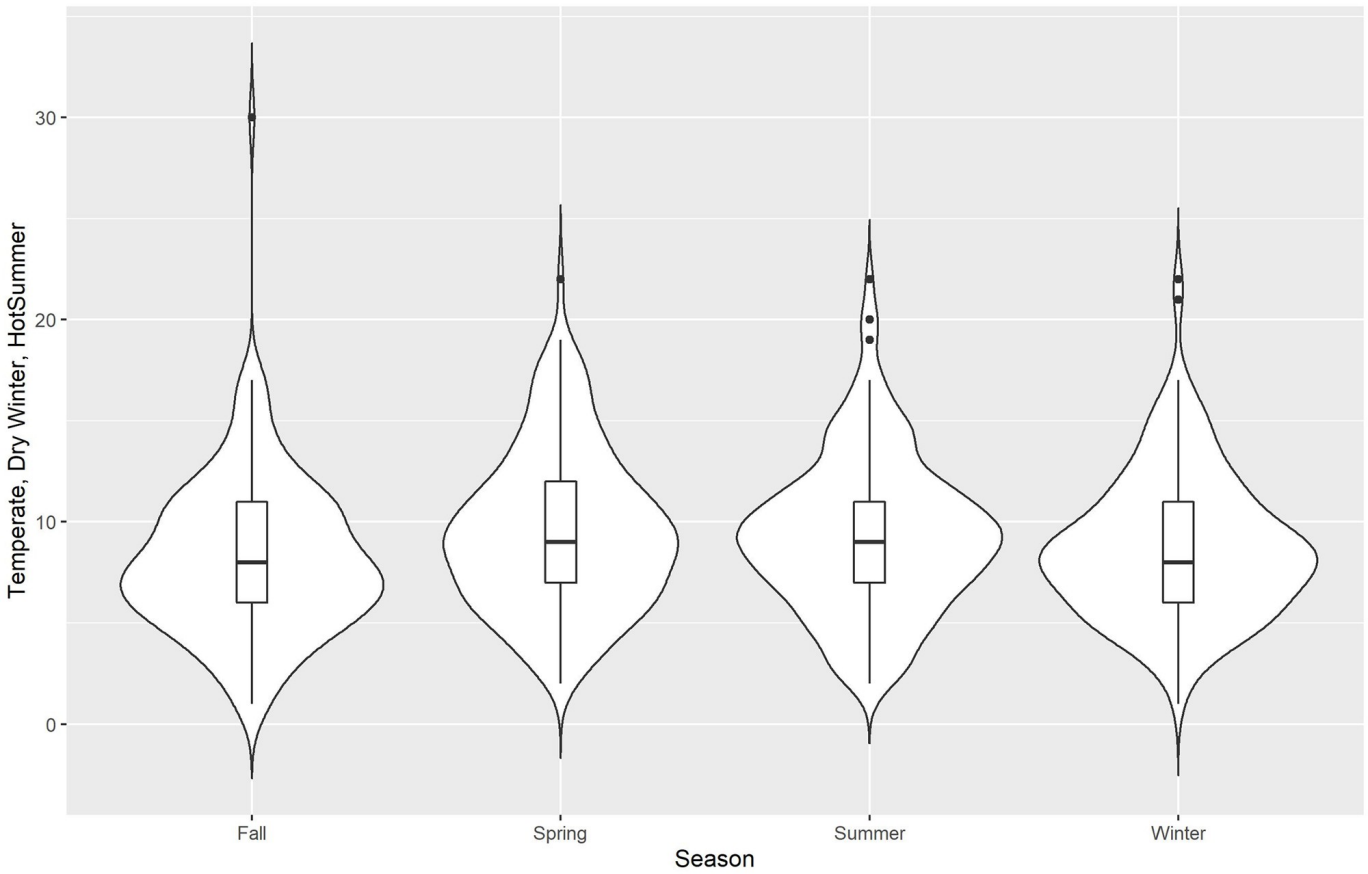
Violin plots for violent crime counts, by season: Temperate, dry winter, hot summer.

**Fig 4:**
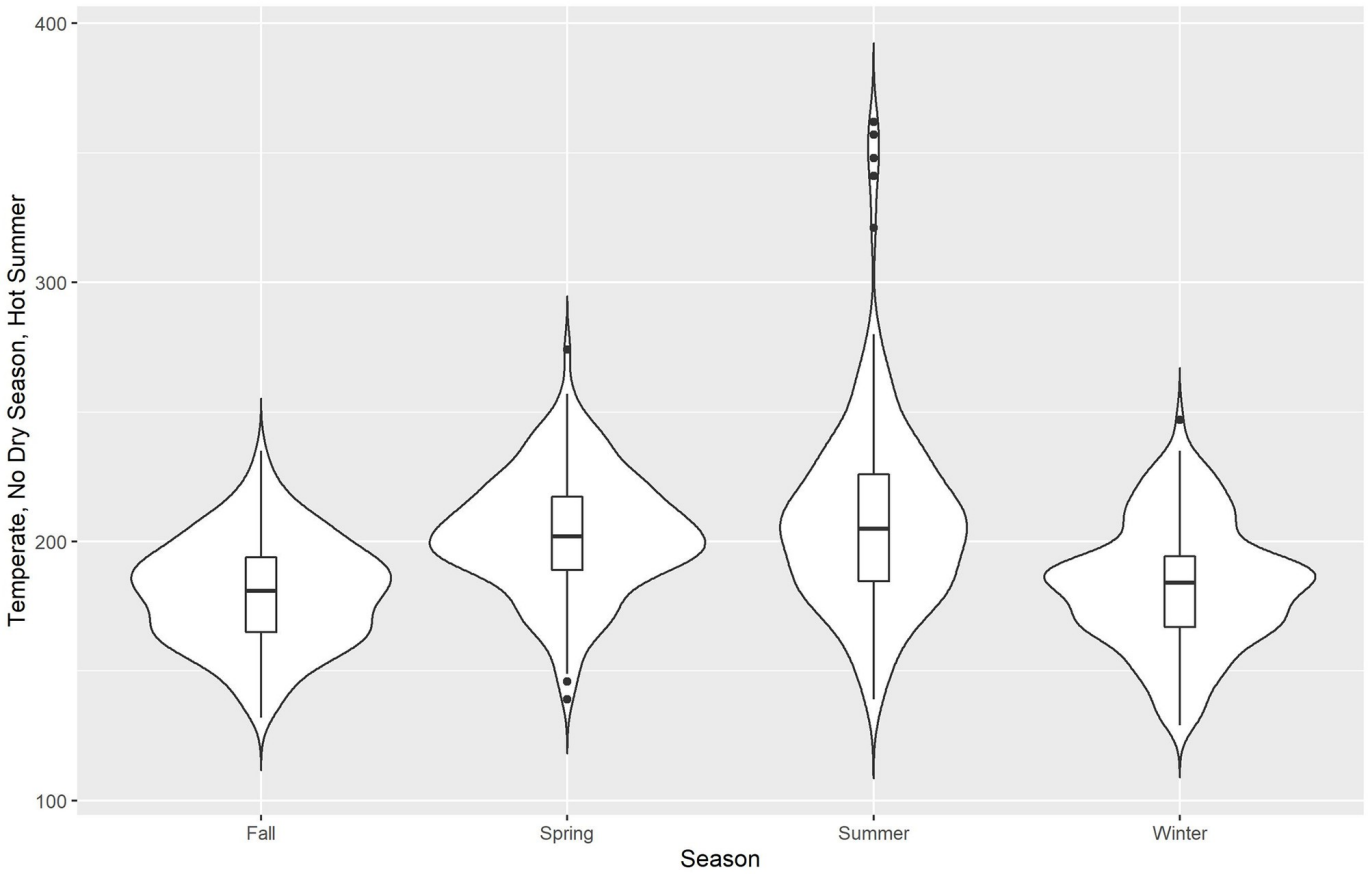
Violin plots for violent crime counts, by season: Temperate, no dry season, hot summer.

**Fig 5:**
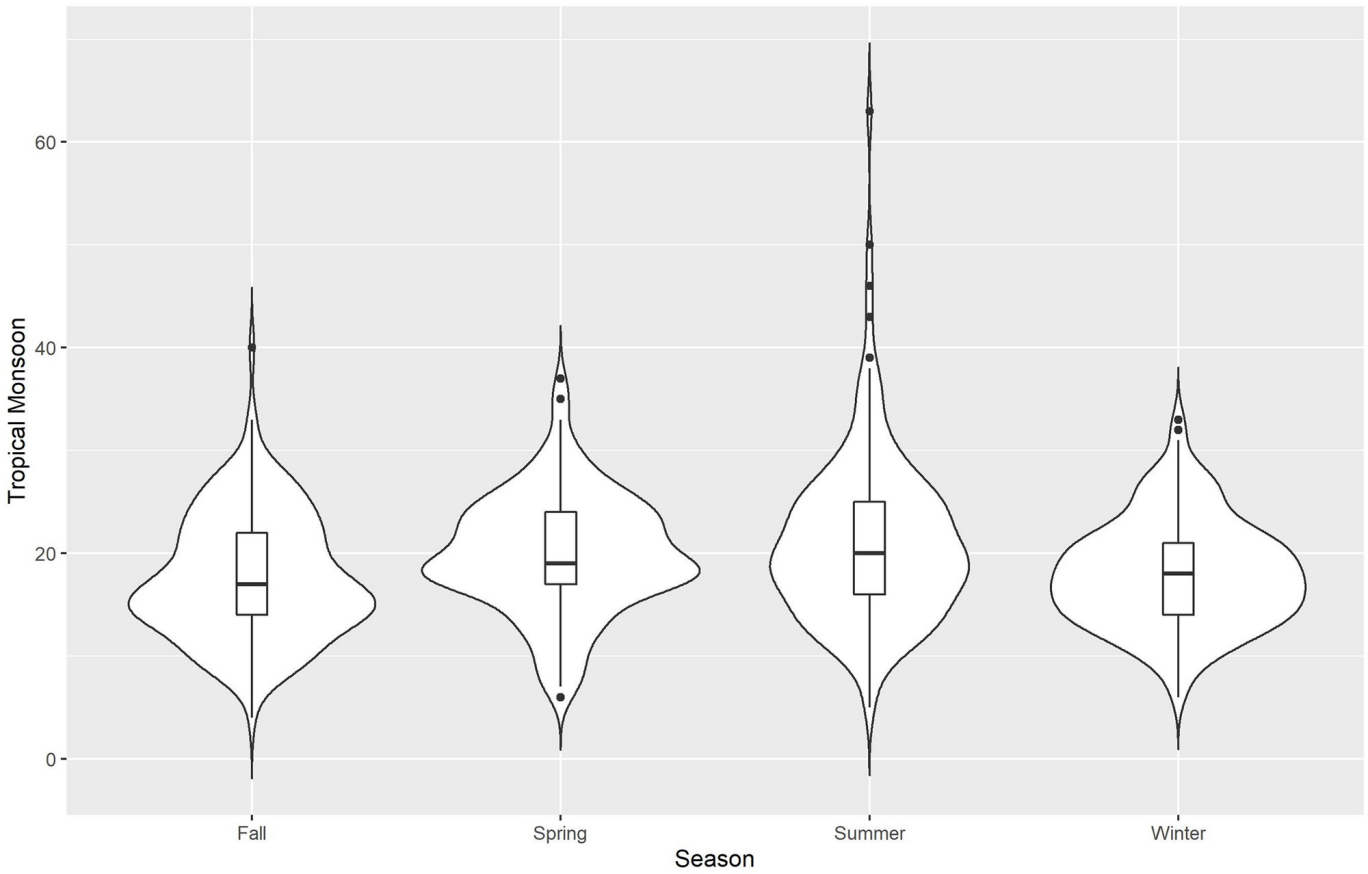
Violin plots for violent crime counts, by season: Tropical monsoon.

**Fig 6:**
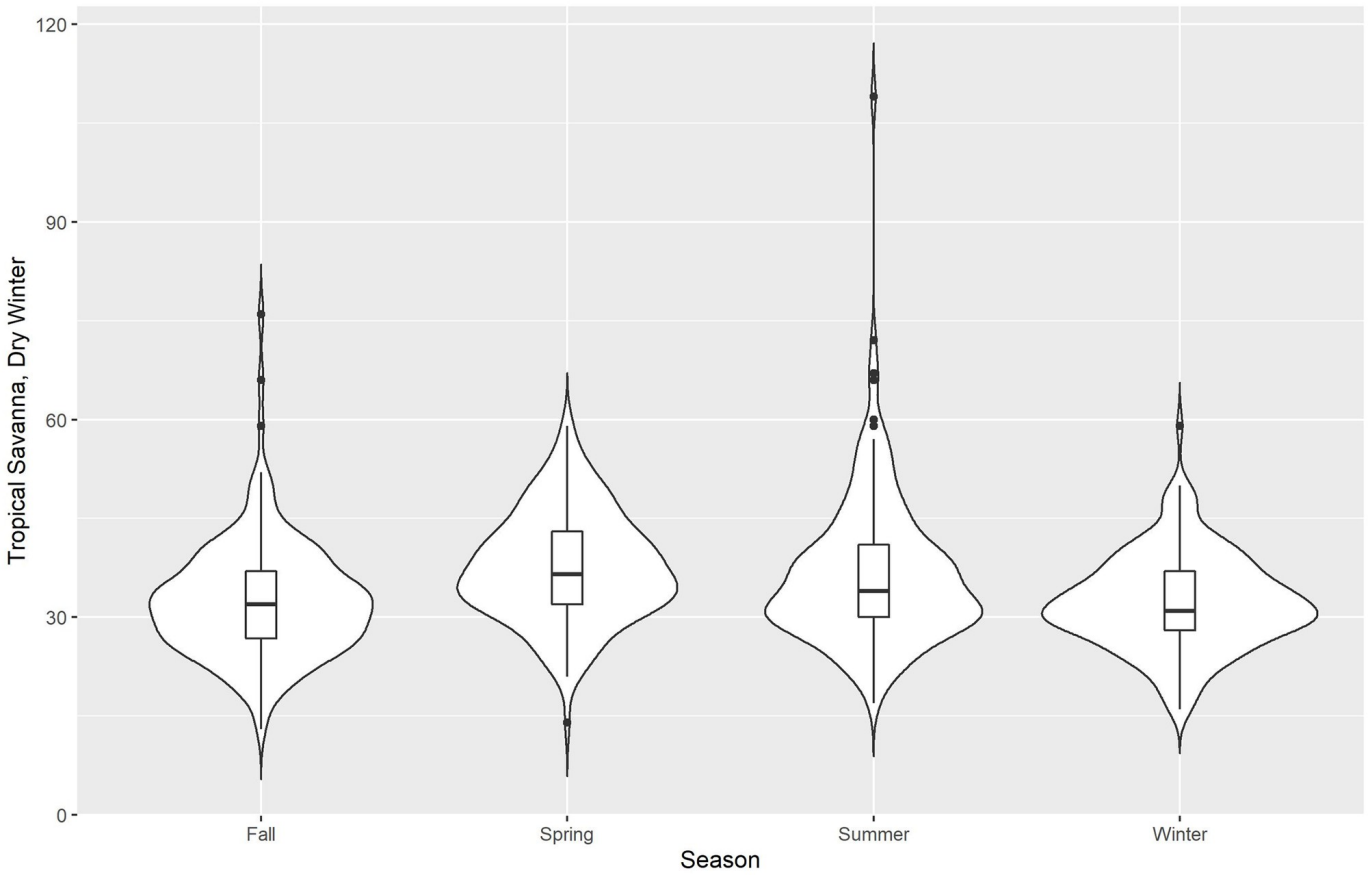
Violin plots for violent crime counts, by season: Tropical savanna, dry winter.

We use violin plots for our initial descriptive presentation of data, Figs 2–6 for the 5 different climate regions in Queensland, Australia. Violin plots are instructive for data visualization because they include the same information as a box plot (this is actually included within the violin plot), plus they show the probability density of the data (violent crime data in the current analyses) at different counts. The violin plots are separated by season, with the wettest time of year in Queensland being summer and fall (October to May)—winter is the driest season.

*Arid steppe hot*, Fig 2, represents violent crime counts for different seasons in the Queensland outback, or western region. This is the driest and hottest climate region in Queensland, considering both the average and maximum values for rainfall and temperature. Fall has the lowest median value and has its highest density of low violent crime counts.

*Temperate*, dry winter, hot summer, Fig 3, generally represents the Mackay region in Queensland, approximately the mid-point up the eastern coast. Similar to arid steppe hot, this climate region has its lowest violent crime counts (by density and by median) during fall.

*Temperate*, no dry season, hot summer, Fig 4, generally represents the Brisbane and Gold Coast areas of Queensland. It has the highest volumes of violent crime simply because of size of the population. Similar to the other two climate regions, the lowest violent crime counts (by density and by median) occur during fall. However, this is also the case for winter in this climate region. As such, as the climate becomes hotter and wetter violent crime counts tend to decrease. Violent crime counts are at their highest levels (by density and by median) during the spring season when temperature is rising but the region is still sufficiently wet (spring begins 01 March in Queensland).

*Tropical monsoon*, Fig 5, as the name implies, is the wettest and one of the hottest climate regions in Queensland—this region contains the popular vacation area of Cairns. Not surprisingly, in this climate region that is closer to the Equator (less variability in climate) the violin plots do not vary dramatically from season to season or in how far the density stretches out. Summer does, however, have the highest counts of violent crime such that hotter and wetter conditions lead to more violence. However, this is also prime vacation time for both Australians and international travel.

Lastly, *tropical savanna*, dry winter, Fig 6, has a similar pattern to tropical monsoon. The violent crime counts (by density and by median) are lowest during fall, but highest during summer. This, again, indicates that violent crime in northern Queensland is at its highest volume at the hottest and wettest time of the year.

These descriptive results generally support both theoretical approaches that are most commonly invoked in the climate and crime literature: temperature aggression theory and the routine activity approach. For temperature aggression theory, violent crimes do increase during the hotter months, but they are also somewhat mediated by the hottest time of the year (aside from some deviations) indicated a curvilinear relationship. In the context of the routine activity approach, there are increases in violent crime counts during vacation times in Queensland, when we expect increases in the number of convergences of motivated offenders and suitable targets.

Turning to the statistical output, Tables 2 to 6 report the negative binomial regression results for the different climate regions in Queensland. We report the raw estimated parameter, the relative risk ratio, and the marginal effect. When comparing across the different climate regions, the relative risk ratios should be considered because of the different base levels of violent crime counts for different climate regions. However, the marginal effects may be instructive for some readers. We consider the relative risk ratios for our interpretations here.

**Table 2:** Negative binomial statistical output: Arid steppe hot.

Variable	Estimate	RRR	Marginal effect	Std. Error	z value	p-value	
Intercept	-0.816	0.442		0.587	-1.390	0.165	
Rain trend	0.112	1.118	0.279	0.028	3.963	< 0.01	***
Rain cycle	-0.003	0.997	-0.007	0.005	-0.594	0.552	
Temperature trend	0.024	1.024	0.059	0.015	1.588	0.112	
Temperature cycle	0.010	1.011	0.026	0.005	1.998	0.046	*
Trend	0.000	1.000	0.000	0.000	4.994	< 0.01	***
Month	0.028	1.028	0.070	0.008	3.484	< 0.01	***
Month-squared	0.001	1.001	0.002	0.000	2.334	0.020	*
Day	0.004	1.004	0.011	0.002	2.542	0.011	*
Day-squared	0.000	1.000	0.000	0.000	-2.434	0.015	*
Monday	0.184	1.202	0.494	0.052	3.567	< 0.01	***
Tuesday	0.292	1.339	0.818	0.049	5.954	< 0.01	***
Wednesday	0.412	1.509	1.205	0.048	8.545	< 0.01	***
Thursday	0.488	1.630	1.470	0.047	10.473	< 0.01	***
Friday	0.386	1.471	1.118	0.050	7.657	< 0.01	***
Saturday	0.264	1.302	0.732	0.051	5.156	< 0.01	***
AIC = 17371							

*Note*. Standard errors, and corresponding p-values, are robust (heteroskedastic and autocorrelation consistent).

**Table 3:** Negative binomial statistical output: Temperate, dry winter, hot summer.

Variable	Estimate	RRR	Marginal effect	Std. Error	z value	p-value	
Intercept	0.250	1.284		0.830	0.301	0.764	
Rain trend	0.005	1.005	0.006	0.011	0.452	0.651	
Rain cycle	0.000	1.000	0.000	0.002	-0.071	0.943	
Temperature trend	-0.009	0.991	-0.012	0.026	-0.350	0.726	
Temperature cycle	0.017	1.017	0.021	0.010	1.690	0.091	.
Trend	0.000	1.000	0.000	0.000	2.082	0.037	*
Month	0.024	1.024	0.030	0.010	2.362	0.018	*
Month-squared	0.001	1.001	0.001	0.000	3.327	< 0.01	***
Day	0.000	1.000	-0.001	0.002	-0.255	0.799	
Day-squared	0.000	1.000	0.000	0.000	0.413	0.680	
Monday	-0.062	0.940	-0.077	0.072	-0.867	0.386	
Tuesday	0.089	1.093	0.117	0.075	1.187	0.235	
Wednesday	0.062	1.064	0.081	0.068	0.921	0.357	
Thursday	0.168	1.183	0.227	0.067	2.516	0.012	*
Friday	0.168	1.183	0.228	0.066	2.540	0.011	*
Saturday	0.046	1.047	0.060	0.067	0.690	0.490	
AIC = 13378							

*Note*. Standard errors, and corresponding p-values, are robust (heteroskedastic and autocorrelation consistent).

**Table 4:** Negative binomial statistical output: Temperate, no dry season, hot summer.

Variable	Estimate	RRR	Marginal effect	Std. Error	z value	p-value	
Intercept	2.268	9.655		0.296	7.660	< 0.01	***
Rain trend	0.006	1.006	0.178	0.008	0.845	0.398	
Rain cycle	-0.003	0.997	-0.078	0.001	-3.968	< 0.01	***
Temperature trend	0.029	1.030	0.813	0.009	3.274	< 0.01	**
Temperature cycle	0.014	1.014	0.396	0.003	5.268	< 0.01	***
Trend	0.000	1.000	0.000	0.000	-3.389	< 0.01	***
Month	0.013	1.014	0.374	0.005	2.995	< 0.01	**
Month-squared	0.002	1.002	0.045	0.000	4.235	< 0.01	***
Day	0.001	1.001	0.035	0.001	1.650	0.099	.
Day-squared	0.000	1.000	0.000	0.000	-1.603	0.109	
Monday	0.004	1.004	0.110	0.017	0.241	0.810	
Tuesday	0.063	1.066	1.800	0.017	3.731	< 0.01	***
Wednesday	0.157	1.170	4.620	0.017	9.373	< 0.01	***
Thursday	0.263	1.301	8.022	0.017	15.811	< 0.01	***
Friday	0.253	1.287	7.671	0.017	14.907	< 0.01	***
Saturday	0.127	1.135	3.673	0.017	7.623	< 0.01	***
AIC = 30460							

*Note*. Standard errors, and corresponding p-values, are robust (heteroskedastic and autocorrelation consistent).

**Table 5:** Negative binomial statistical output: Tropical monsoon.

Variable	Estimate	RRR	Marginal effect	Std. Error	z value	p-value	
Intercept	-0.103	0.902		0.513	-0.201	0.840	
Rain trend	0.000	1.000	-0.001	0.004	-0.065	0.948	
Rain cycle	0.000	1.000	0.000	0.000	-0.281	0.779	
Temperature trend	-0.001	0.999	-0.004	0.015	-0.085	0.933	
Temperature cycle	0.005	1.005	0.014	0.005	0.953	0.341	
Trend	0.000	1.000	0.000	0.000	0.310	0.757	
Month	0.335	1.398	0.915	0.017	19.538	< 0.01	***
Month-squared	-0.007	0.993	-0.019	0.001	-6.014	< 0.01	***
Day	0.001	1.001	0.003	0.001	1.071	0.284	
Day-squared	0.000	1.000	0.000	0.000	-0.891	0.373	
Monday	-0.003	0.997	-0.007	0.025	-0.109	0.913	
Tuesday	0.029	1.029	0.080	0.028	1.054	0.292	
Wednesday	0.045	1.046	0.126	0.028	1.597	0.110	
Thursday	0.037	1.038	0.102	0.026	1.402	0.161	
Friday	0.027	1.027	0.075	0.026	1.026	0.305	
Saturday	0.032	1.032	0.087	0.032	0.983	0.325	
AIC = 12586							

*Note*. Standard errors, and corresponding p-values, are robust (heteroskedastic and autocorrelation consistent).

**Table 6:** Negative binomial statistical output: Tropical savanna, dry winter.

Variable	Estimate	RRR	Marginal effect	Std. Error	z value	p-value	
Intercept	0.188	1.207		0.841	0.224	0.823	
Rain trend	-0.014	0.987	-0.067	0.008	-1.789	0.074	.
Rain cycle	-0.001	0.999	-0.005	0.002	-0.389	0.697	
Temperature trend	0.045	1.046	0.222	0.026	1.741	0.082	.
Temperature cycle	0.023	1.023	0.114	0.010	2.385	0.017	*
Trend	0.000	1.000	0.000	0.000	-0.757	0.449	
Month	0.003	1.003	0.014	0.007	0.415	0.678	
Month-squared	0.002	1.002	0.010	0.000	4.849	< 0.01	***
Day	0.000	1.000	-0.001	0.001	-0.208	0.835	
Day-squared	0.000	1.000	0.000	0.000	0.109	0.913	
Monday	-0.055	0.947	-0.266	0.035	-1.550	0.121	
Tuesday	0.063	1.065	0.321	0.040	1.593	0.111	
Wednesday	0.161	1.175	0.850	0.036	4.496	< 0.01	***
Thursday	0.227	1.254	1.224	0.035	6.447	< 0.01	***
Friday	0.243	1.275	1.318	0.035	6.974	< 0.01	***
Saturday	0.168	1.182	0.886	0.043	3.928	< 0.01	***
AIC = 21230							

*Note*. Standard errors, and corresponding p-values, are robust (heteroskedastic and autocorrelation consistent).

*Arid steppe hot*, Table 2, reports the most statistically significant parameter estimates. For the control variables, the overall trend and seasonal variables are all positive and statistically significant. This is not surprising for the overall trend and month-squared variables (because Queensland is in the Southern Hemisphere the seasonal variables are expected to be negative and positive for the linear and squared terms, respectively), but month is expected to be negative. This could simply be because there is not climatic variation that leads to variations in violent crime over the year, but violence is just increasing in this region of Queensland. With regard to the variables of interest (rainfall and temperature), rain trend and temperature cycle have positive relationships with violent crime counts, increasing by 11.8 and 1.1 percent, respectively, with one-unit increases.

*Temperate*, dry winter, hot summer, Table 3, has the same statistical results for the overall trend and seasonality variables as *arid steppe hot*, when statistically significant. However, only Thursday and Friday have statistically significant increases relative to the baseline (Sunday), at 18.3 percent. And the climate variables are statistically insignificant aside from temperature cycle having a positive and marginally significant relationship with violent crime counts.

*Temperate*, no dry season, hot summer, Table 4, represents the statistical results for Brisbane, the Gold Coast, and surrounding areas. The control variables for trend and seasonality, when statistically significant, have the same relationship as the other two climate regions (increasing). This most definitely indicates that seasonality does not operate in the same manner as in the cooler Northern Hemisphere studies. All of the days of the week aside from Monday have statistically significant increases in violence compared to the baseline. The largest magnitude relative risk ratios are for Thursday and Friday, the latter being expected. The climate variables indicate that increases in temperature (in trend and the deviations from trend) lead to increases in violence, consistent with theoretical expectations. The trend in rain is not statistically significant, but deviations in rainfall trend (increases) lead to decreases in violence: every 1-millimetre increase in rain over trend leads to a 7.8 percent decrease in violent crime counts.

*Tropical monsoon*, Table 5, representing the area around Cairns, exhibits the fewest statistically significant results. Only month and month-squared are statistically significant, positive and negative, respectively. This is opposite expectations given the seasonal cycle in Queensland, but may be due to the relatively cooler temperatures allowing for more convergences of motivated offenders and suitable targets in outside spaces. And though statistically insignificant, the estimated parameters for rainfall and temperature do indicate that increases in those variables are related to decreases in violent crime counts.

Lastly, *tropical savanna*, dry winter, Table 6, generally representing the northern region of Queensland shows increases in violent crime counts on Wednesday, Thursday, Friday, and Saturday. Month-squared is also positive and statistically significant. With regard to the climate variables, increases in temperature (trend and cycle) lead to increases in violent crime counts, albeit relatively small;4.6 and 2.3 percent, respectively. Increases in rainfall trend, however, does lead to decreases in violent crime counts.

## Discussion

In our analyses, we consider the impact of weather on crime in a longitudinal study (2008–2019, 12 years) of daily patterns, across 7 Köppen climate regions (we analyzed 5, aggregating 2 of them because of data limitations), considering both trends and deviations in trends for temperature and rainfall across all of Queensland, Australia. These analyses have revealed a number of interesting results that allow for greater generalizability given the broad scope of our research design.

First, examining just our control variables, we find no evidence for a fall in crime (violence) during the 12-year time period. Though the international (violent) fall in crime has varied in both timing and magnitude [61–65], no meaningful drop in violent crime is detected in the current study. A negative and statistically significant estimated parameter was found in the Brisbane–Gold Coast region (temperate, no dry season, hot summer), but the associated trend is effectively zero. This has important implications for theory and policy. If violence is increasing in Queensland, despite international declines (or even stability in violent crime), more research is necessary to determine why this is occurring and how to prevent further increases.

Second, given that Queensland, Australia is in the Southern Hemisphere, any seasonal pattern is expected to be U-shaped because winter is in the middle of the year. As such the estimated parameters for month and month-squared are expected to be negative and positive, respectively. We only find this result in the Cairns region (tropical monsoon), with the other Köppen regions having positive estimated parameters for both seasonality variables. This is an important result because it demonstrates that seasonality is not ubiquitous across regions and highlights the importance of analyzing these regions separately. Rather, we see that seasonality has a geography and violent crime is dependent on where you are investigating, even in a largely subtropical region of the world.

Third, considering the weather-related variables, the overall result is that increases in rain lead to decreases in violence and increases in temperature lead to increases in violence. This is consistent with the extant literature, demonstrating support for the routine activities perspective in diverse climate regions [15–19]. Though a positive relationship between temperature and violence is consistent with temperature aggression theory, considered together, the impact of deviations in heat AND precipitation better support the routine activities perspective. Poorer weather conditions (more rain) lead to fewer convergences of motivated offenders and suitable targets outside the home, and warmer conditions (that are related to vacation and school schedules in Queensland, Australia) lead to increases in those convergences. This is further supported by the day of the week variables that indicate that unstructured time, like at the end of week, create more opportunities for crime.

Lastly, the strongest predictors in our models (based on the magnitude of the relative risk ratios and the marginal effects) are the days of the week. Violence increases as the week progresses, peaking on Thursday and Friday in most Köppen climate regions. The only regions that do not exhibit this pattern is the tropical monsoon region (Cairns), a vacation area whose inhabitants are more likely to be tourists with more unstructured free time.

Overall, with the results presented here, we find support for the routine activities perspective. We find a positive relationship between temperature and violent crime and a negative relationship between precipitation and violent crime. Moreover, the magnitude of impact from the day of the week variables (that represent the routine activities perspective) are the strongest when considering all of the variables in our statistical models. As such, is instructive for understanding temporal patterns of crime in a non-temperate, Southern Hemisphere context.

## Limitations and future directions

Despite the instructive results, our analyses are not without their limitations. First, our analyses rely on police-reported data. These data have a well-known and long-standing lack of reporting [79–81]. However, reporting of crime to the police in Australia is higher than many western countries, particularly for violence: 54% (assault), 39% (sexual assault), 90% (theft of vehicle), 75% (residential burglary) [82]. As such, though crime reporting is an issue, we use 12 years of data such that any reporting issues should be consistent across our longitudinal analyses, and this is a lesser issue in the context of Australian police data. Future studies may consider exploring victimization or self report data if it is rigorous and representative, in order to better understand violence that is not captured by police-reported data.

We also do not include property crime. This exclusion is not only beyond the scope of the current analyses, but it allows us to delve deeper into the relationships between weather and violence. Moreover, temperature aggression theory is a theory that relates to violence, so the current analysis allows us to test across theories. However, future research should continue to explore multiple crime types across several different regions.

We also we not able to directly explore the impact of humidity for its relationship with violence because of data availability. Previous research has shown that humidity and various forms of violence have a negative relationship [10, 49]. We do, however, use Köppen climate regions that control for humidity, so this factor is controlled for indirectly in our study. Future studies may want to control for humidity directly, if access to this data is available.

Though not a limitation, per se, it is important to note that we only analyze daily data, not accounting for variations within the day [56]. Because of this, all interpretations must be in that context, not making any inferences for changes in temperature and rain within the day. Lastly, we do not control for region-specific socio-economic factors such as the economy and population growth. However, these factors are indirectly accounted for, similar to above, through the analysis of separate climate regions of Queensland and longer-term trend and seasonality variables.

## Conclusion

The impact of climate change on the modern world is substantial. As climate extremes, or weather shocks increase in frequency and intensity, opportunities for crime will continue to change. In this study, we explored the relationship between heat, precipitation, and violent crime across several climate regions in the state of Queensland, Australia. We found that, increases in heat and precipitation have opposite impacts on violent crime and this finding holds across each climate regions. This has important policy implications. Policy makers and police can better prepare for changes in violence when major weather fluctuations are expected. Indeed, as climate change continues to create weather extremes, the implications for crime opportunities are substantial. Policy makers will need to prepare health care workers and first responders for increasing workloads, and crime prevention strategies will need to consider how weather shapes the location and timing of crime opportunities.
